# Supplementary material for: Regional cooling caused recent New Zealand glacier advances in a period of global warming
Source: Nat Commun. 2017 Feb 14;8:14202. doi: 10.1038/ncomms14202 (PMC5316876; doi:10.1038/ncomms14202)
Supplement: Supplementary Information — Supplementary Figures, Supplementary Tables, Supplementary Discussion and Supplementary References. [file ncomms14202-s1.pdf]

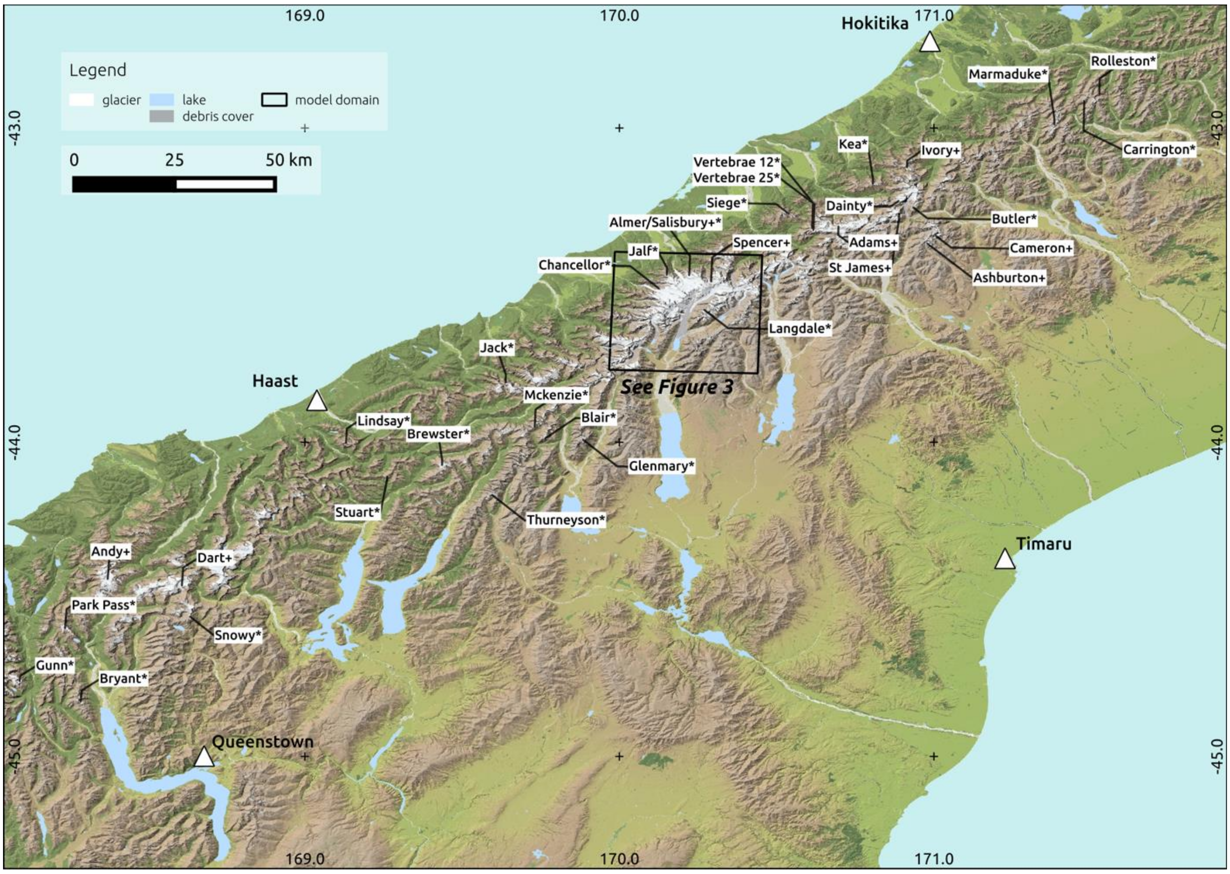

Supplementary Figure 1. Map of the central South Island of New Zealand, showing the location of Southern Alps glaciers. The glaciers that are marked with an asterisk (*) are used to derive annual snowline departures (see reference[1] for details). Glaciers marked with a plus sign (+) advanced for at least five of the years between 1983 and 2008[2]. The mass balance model domain (Figure 3) is also marked. This figure was generated using QGIS[3].

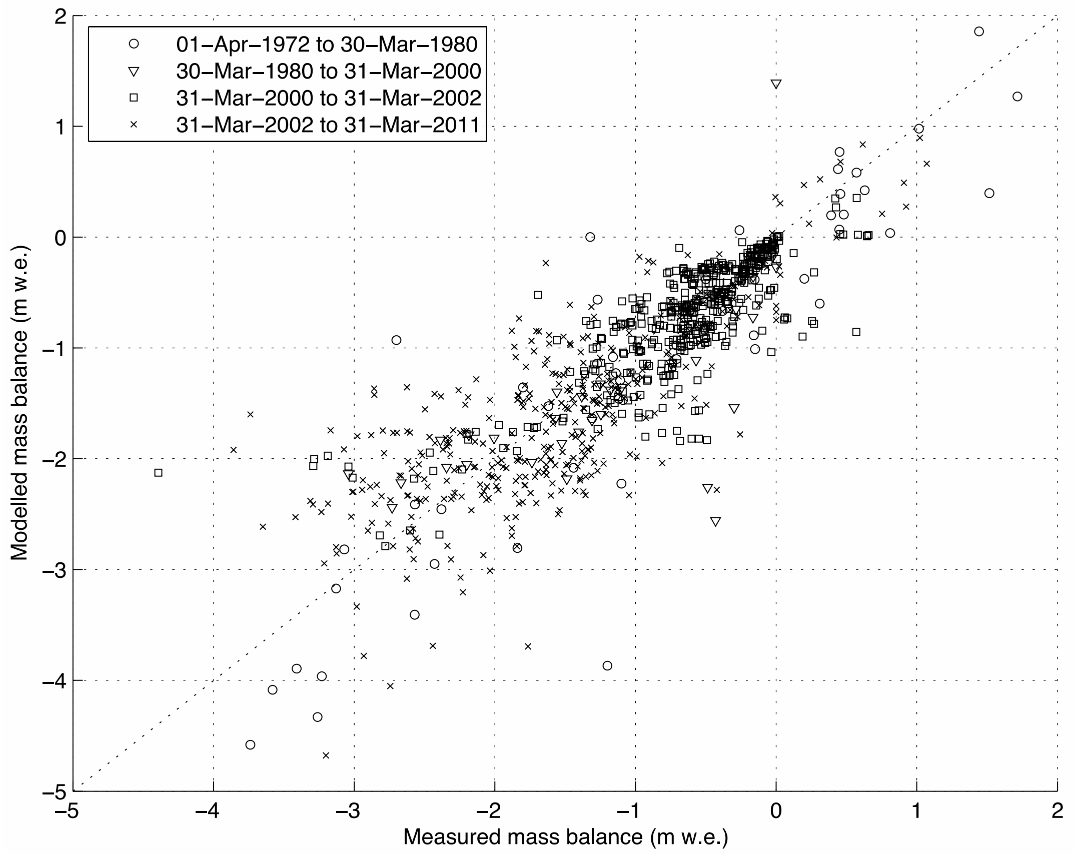

Supplementary Figure 2. Testing and evaluation of our glacier mass balance model in scatter plot for modelled versus measured mass balance. The scatter plot shows glacier mass balance data in metres of water equivalent (m w.e.) from Tasman and Franz Josef glaciers (Supplementary Table 2). Symbols show the tuning (2000-2002) and testing periods (others). A 1:1 line is shown. Correlation coefficient (r) values for each of the datasets are provided in Supplementary Table 2.

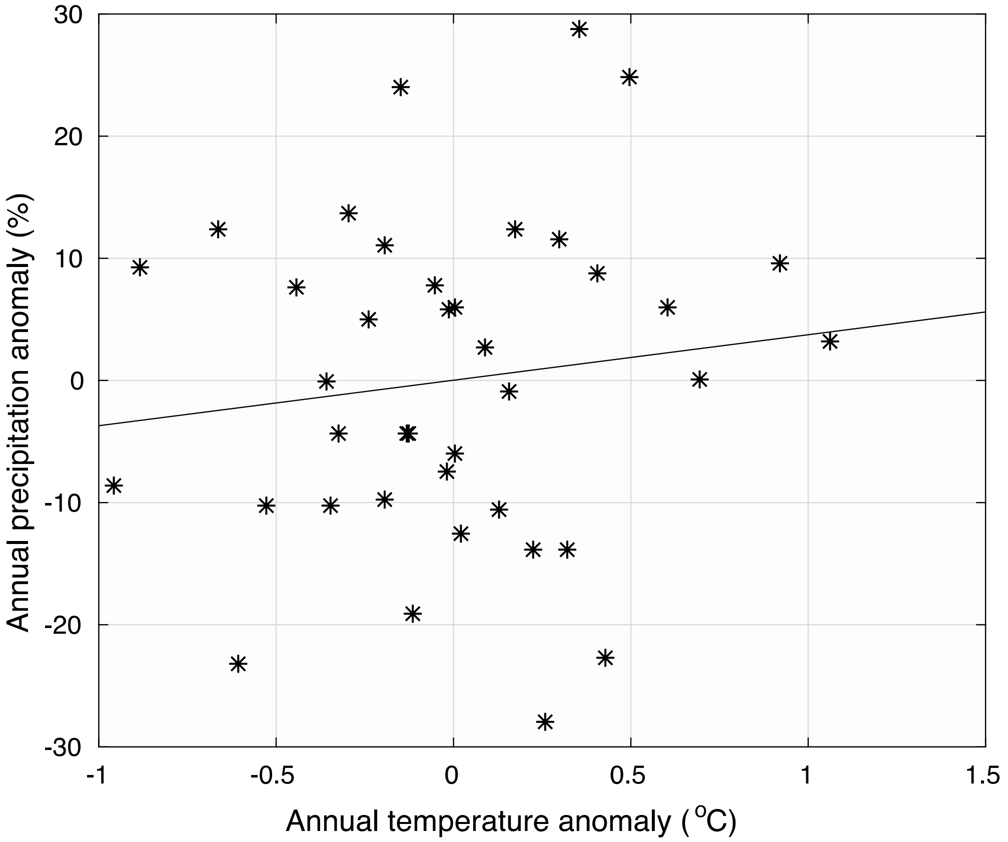

Supplementary Figure 3. Scatter plot of annual mean temperature and precipitation anomalies over our model grid. The solid line shows the regression between temperature and precipitation (r=0.12, p >0.1). This figure shows that these two variables are uncorrelated within our model domain and simulation period.

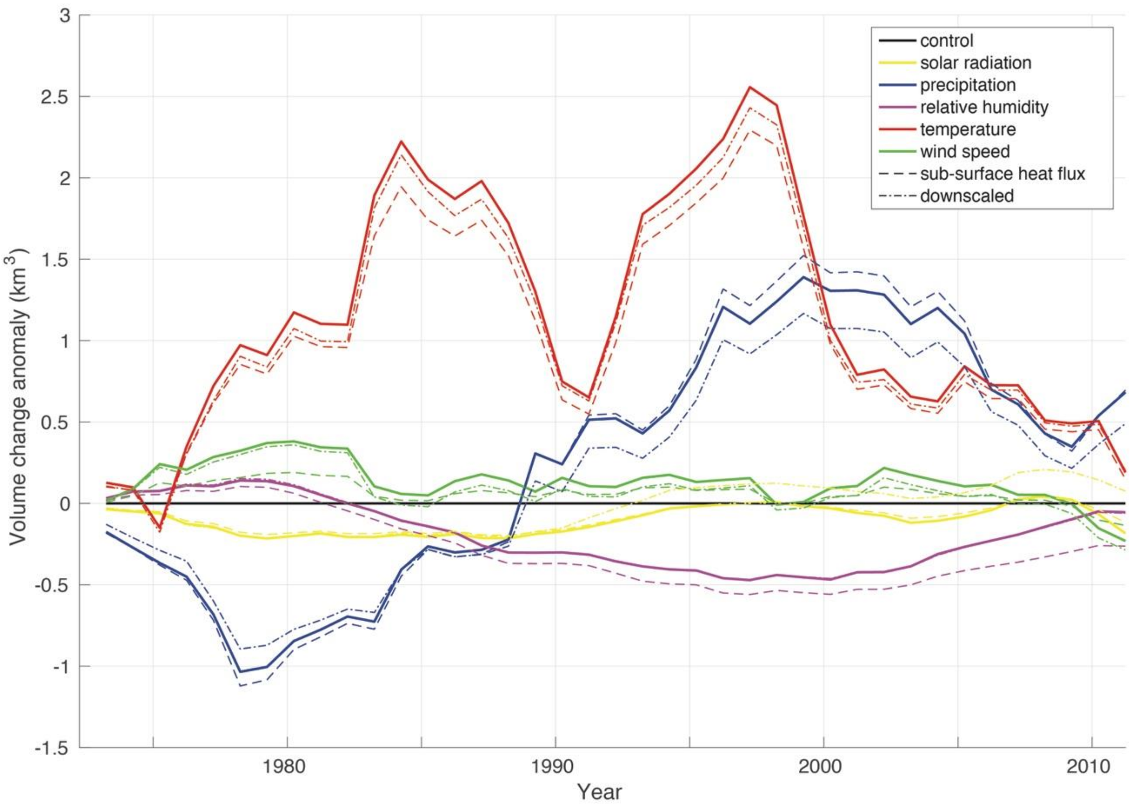

Supplementary Figure 4. Model experiments showing the influence of two different model assumptions: (dash-dot lines) using downscaled temperature input data; and (dashed lines) the sub-surface thermal calculations which include heat conduction under debris cover.

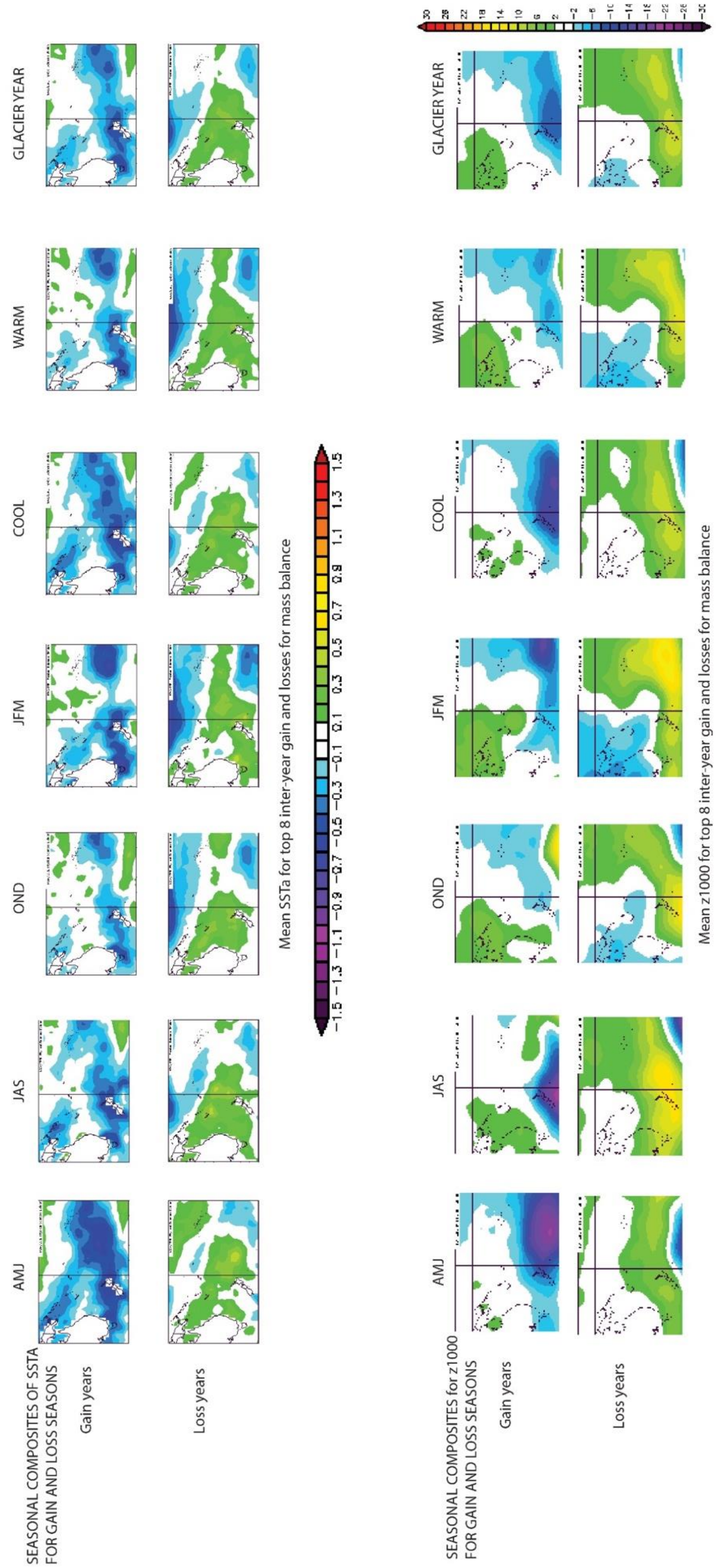

Supplementary Figure 5 (caption on next page).

Composite plots of upper and lower quintile seasons/years for sea surface temperature (SSTa) and geopotential height anomalies (1000hPa). This figure shows the seasonal components of the atmospheric and oceanographic conditions that promote glacier mass gain and mass loss in the Southern Alps. Anomalies are relative to the 1981-2010 period. SST scale (-1.5 to 1.5) is in degrees Celsius (ºC), while geopotential anomalies (-30 to 30) are in metres. Additional anomaly plots for sea ice concentration, a Southern Hemisphere polar stereographic view of z1000, precipitable water content (PWC) and the individual seasons or years that comprise these composites can be found at https://speakerdeck.com/spclimateniwa/composite-plots-for-glacier-mass-balance-gain-and-mass-balance-loss-seasons-and-years. Data/images provided by the NOAA/OAR/ESRL PSD, Boulder, Colorado, USA, from their Web site at http://www.esrl.noaa.gov/psd/

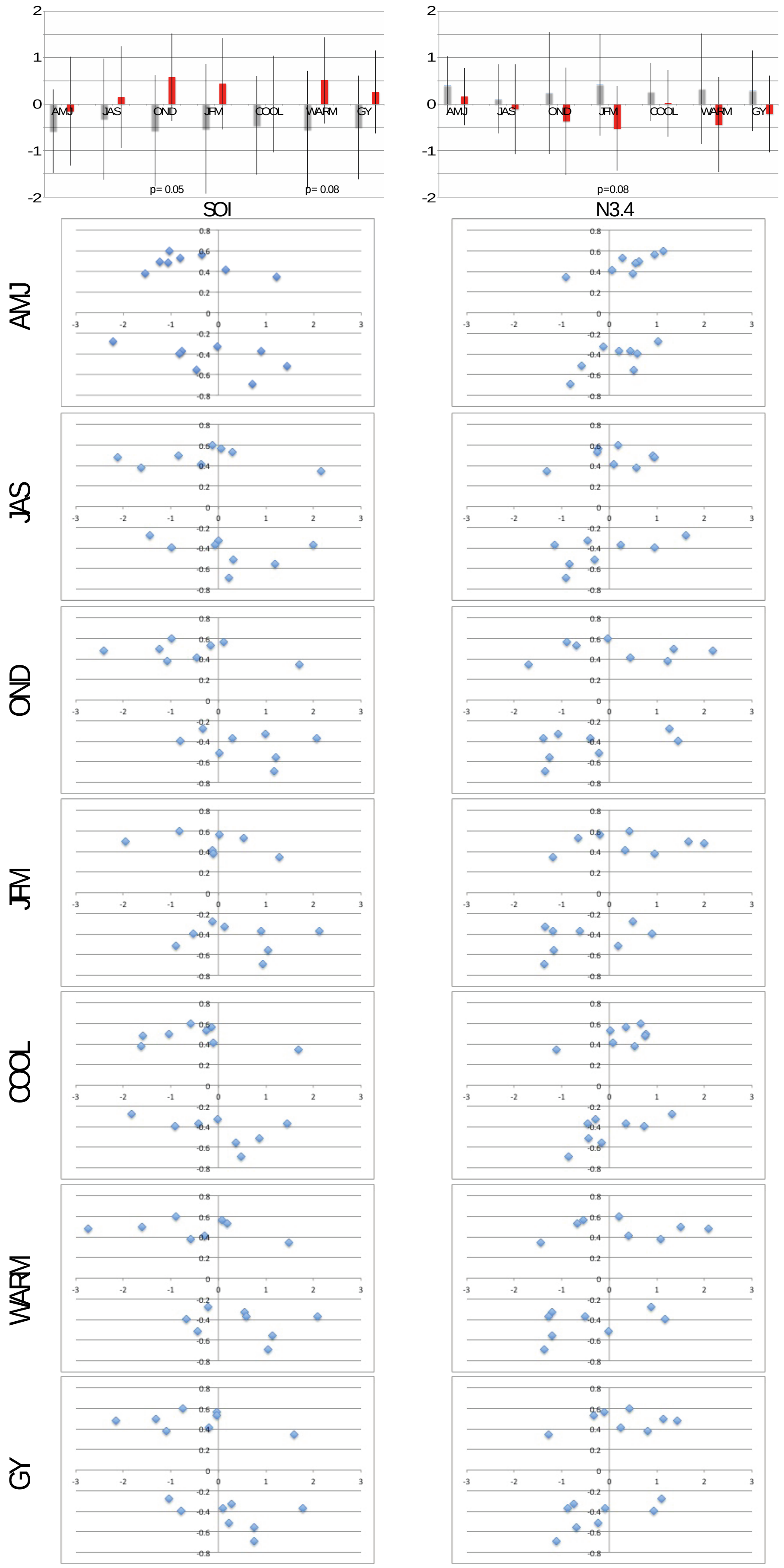

Supplementary Figure 6 (caption on next page).

The histogram and whisker plot shows the mean and standard deviation for mass balance gain (blue) and loss (red) that correspond to the Southern Oscillation Index (SOI) or Nino3.4 (N3.4) values for different times of the glacier year. Scatter plots of individual mass balance values that comprise the means (shown by each histogram) versus individual SOI (left) and N3.4 values (right) are shown for all seasons. For each scatter plot, the *x* axis is the climate index value, while the *y* axis is the simulated glacier mass balance change (km^3). No strong linear relationships are observed for any season.

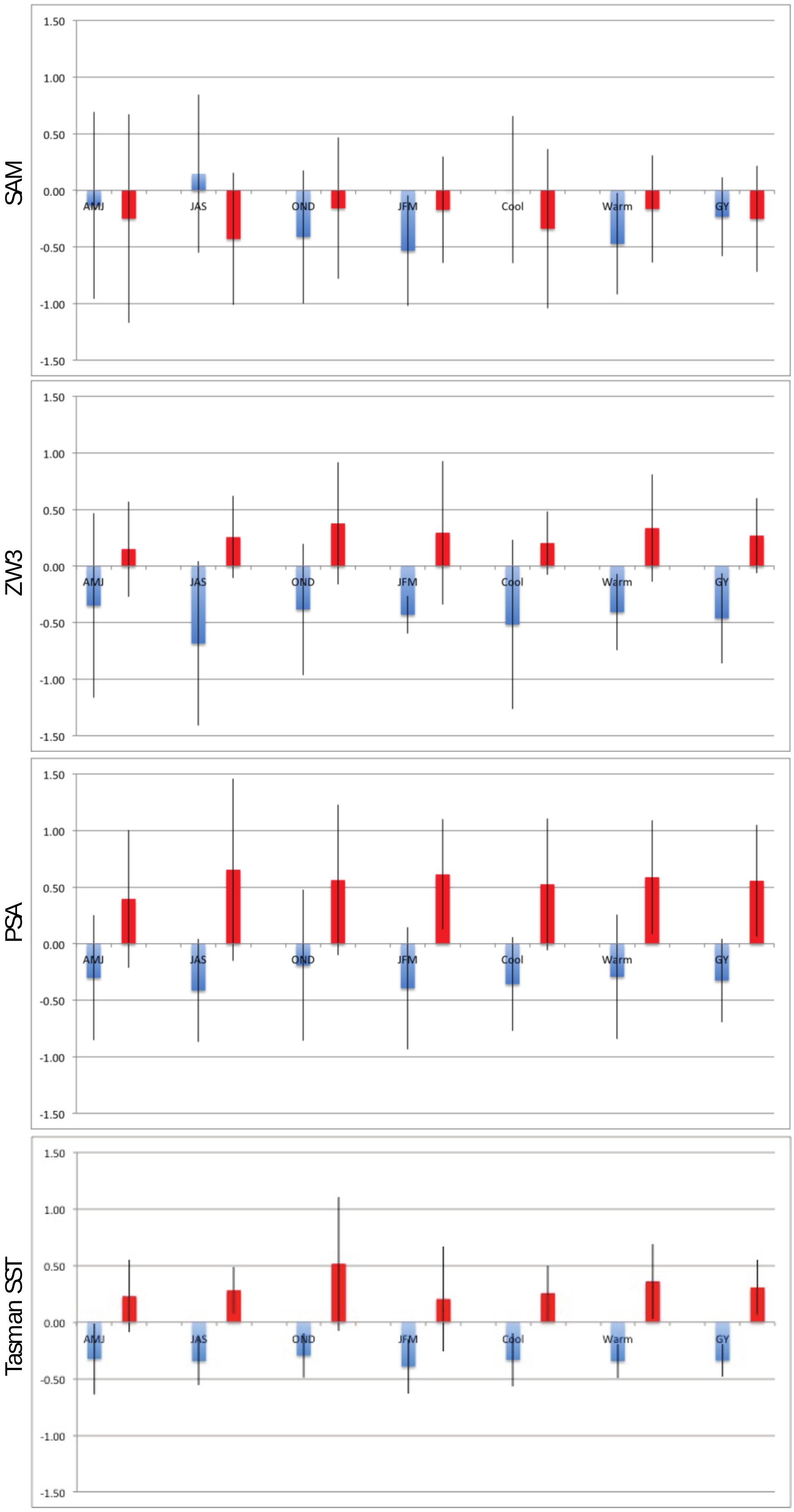

Supplementary Figure 7 (caption on next page).

Relationship between Southern Annular Mode (SAM) index, Zonal Wave 3 (ZW3) index, Pacific South American pattern (PSA) index, Tasman Sea surface temperature (SST) anomaly and glacier volume changes for seasons and glacier year. Y axes denote index value anomalies for SAM, ZW3 and PSA, while temperature anomalies (°C) are shown for the Tasman Sea. SAM, ZW3 and PSA index anomaly values were calculated relative to the 1981-2010 climatological normal interval using reanalysis data[4]. Both ZW3 and PSA are based on 500hPa geopotential height; ZW3 is calculated per reference[5] and the PSA index used is the amplitude time series of the second Empirical Orthogonal Function of monthly mean 500hPa geopotential height over the Southern Hemisphere south of 20°S. The SAM index is calculated using normalised monthly zonal mean sea level pressure difference between 40°S and 65°S following reference[6].

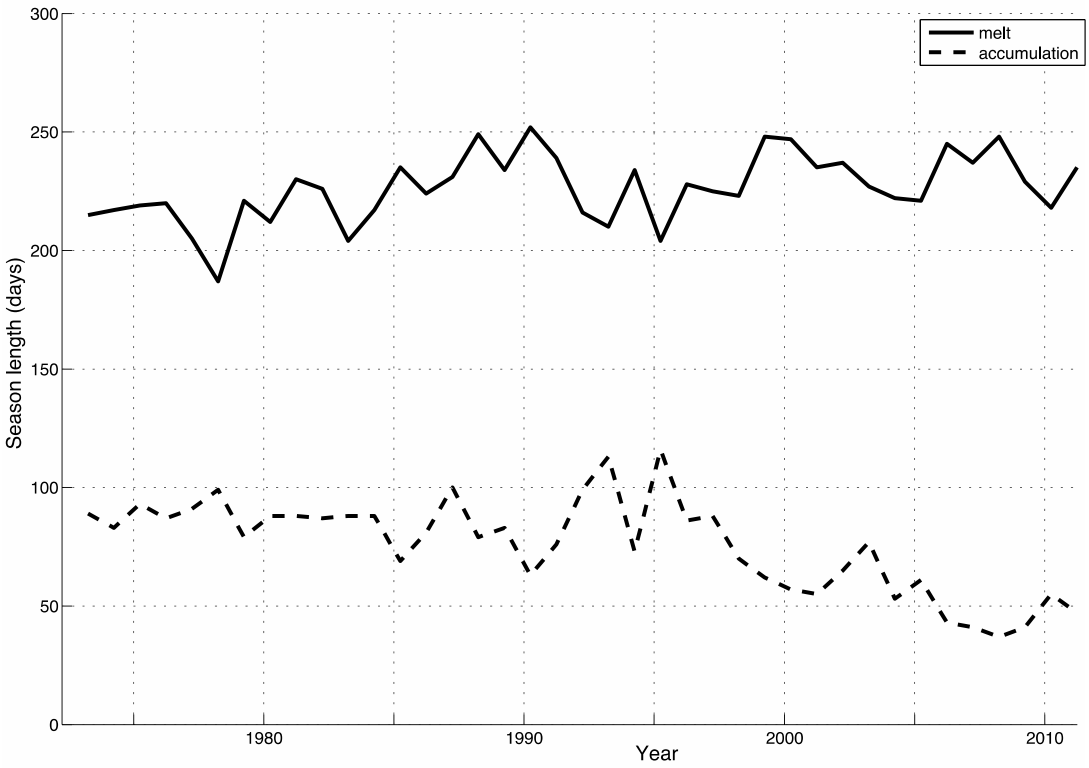

Supplementary Figure 8. Simulated length of the accumulation and ablation seasons over the model grid in the ‘standard’ model run. Ablation season length was calculated as the total number of days when net ablation occurred, and accumulation season length as total number of days when net accumulation occurred. As season length varies with elevation, we selected a site near the long-term equilibrium line of Tasman Glacier (1740 m above sea level) to examine changes in the length of the accumulation and ablation seasons.

Supplementary Table 1: A summary of the values of parameters used to calculate model input data and mass balance. Minimum and maximum values are used to test model sensitivity to parameters with uncertain values. Sensitivity to albedo characteristic depth and time scale were not tested because previous work has shown the mass balance simulation to be insensitive to these parameters. An addition sensitivity test was carried out by increasing the inferred cloudiness value by +/- 0.05 recognising the uncertainty in estimating cloudiness over this mountainous domain.

Parameter	Symbol	Value	Minimum value	Maximum value	Source
**Climatic variations**					
temperature lapse rate	$\frac{dT}{dh}$	Seasonally variable	4°C/km	6°C/km	[7]
**Shortwave radiation**					
albedo of fresh snow	$\alpha_{fresh\ snow}$	0.9	0.85	0.95	[8]
albedo of firn	α_{firn}	0.53	0.48	0.58	[8]
albdeo of ice	α_{ice}	0.36	0.31	0.41	[8]
albedo characteristic depth	d_c	11 mm w.e.			[8]
albedo characteristic time scale	t_c	21.9 days			[8]
**Turbulent heat fluxes**					
roughness parameter for ice	z_{ice}	0.004 m	0.002 m	0.006 m	Tuned to Franz Josef ablation measurements 2000-2002
roughness parameter for snow	z_{snow}	0.0004 m	0.0002 m	0.0006 m	Tuned to Franz Josef ablation measurements 2000-2002
**Accumulation**					
snow/rain temperature threshold	T_s	1 ºC	0 ºC	2 ºC	[9]

Supplementary Table 2: A summary of the mass balance data used in this study. Data for model tuning are taken from Franz Josef Glacier in the 2001 and 2002 balance years, while all other data provide an independent test of model performance.

Purpose	Glacier	Balance year	Number of observations	r
Testing	Tasman Glacier[10]	1 April 1972 to 31 March 1975	54	0.92
Testing	Tasman Glacier[11]	November 1985 to May 1987	35	0.73
Tuning	Franz Josef Glacier[9]	1 April 2000 to 31 March 2002	455	0.81
Testing	Franz Josef Glacier[9]	1 April 2002 to 31 March 2010	288	0.77
Testing	Tasman Glacier[12]	1 April 2007 to March 2009	65	0.86

Supplementary Table 3: 'Snowline' glaciers used in model evaluation (Figure 5), including location reference and base Equilibrium Line Altitude (ELA_0).

**Glacier**	**New Zealand Glacier Inventory[13] number, Randolf Glacier Inventory (RGI) number[14]**	**ELAo**
Mt. Wilson Glacier	None	1912
Rolleston Glacier	911A/004, RGI32-18.02910	1763
Mt. Carrington Glacier	646C/027, RGI32-18.02901	1715
Marmaduke Glacier	664C/012, RGI32-18.02823	1830
Mt. Butler Glacier	685C/060, RGI32-18.03141	1840
Dainty Glacier	897/019, RGI32-18.03075	1954
Kea Glacier	897/007, RGI32-18.03385	1820
Siege Glacier	893A/006, RGI32-18.03303	1736
Vertebrae #12 Glacier	893A/012, RGI32-18.03213	1864
Vertebrae #25 Glacier	893A/025, RGI32-18.03216	1840
Langdale Glacier	711I/035, RGI32-18.02452	2186
Salisbury Glacier	888B/003, RGI32-18.03474	1810
Jalf Glacier	886/002, RGI32-18.02456	1790
Chancellor Dome	882A/007, RGI32-18.02503	1756
Glenmary Glacier	711F/006, RGI32-18.01625	2175
Blair Glacier	711D/038, RGI32-18.01534	1938
Mt Mckenzie Glacier	711D/021, RGI32-18.01994	1904
Jack Glacier	875/015, RGI32-18.01669	1907
Thurneyson Glacier	711B/012, RGI32-18.01382	1970
Brewster Glacier	868C/020, RGI32-18.01130	1935
Mt. Stuart Glacier	752I/104, RGI32-18.01466	1673
Lindsay Glacier	867/002, RGI32-18.01150	1730
Snowy Ck Glacier	752C/103, RGI32-18.01032	2092
Park Pass Glacier	752B/048, RGI32-18.00569	1824
Bryant Glacier	752B/025, RGI32-18.00448	1783
Mt. Gunn Glacier	851B/057, RGI32-18.00375	1593

Supplementary Table 4: Upper (positive, mass gain) and lower (negative, mass loss) quintile years for modeled glacier mass balance volume change anomalies. 'Glacier year' represents the period 1 April to 31 March (of following year). The full dataset of glacier volume anomalies is plotted in Figure 5A. This table also shows the corresponding anomalies in temperature, precipitation, shortwave radiation, relative humidity, and wind speed for each listed glacier year.

Ranking	Glacier Year	Volume Change (km^3)	Temperature anomaly (K)	Precipitation anomaly (%)	Shortwave radiation anomaly (%)	Relative humidity anomaly (%)	Wind speed anomaly (%)
Mass gain years							
1	1993	0.5989	-0.92	-9.65	-5.04	1.43	-5.05
2	1984	0.5608	-0.43	22.68	-2.75	2.83	3.44
3	1996	0.5304	-0.26	27.91	-1.55	2.35	-0.99
4	1992	0.4939	-0.69	-0.06	-7.56	2.12	2.77
5	1983	0.4831	-1.06	-3.2	-0.01	2.12	14.14
6	1980	0.4161	-0.32	13.93	-2.97	1.5	-0.9
7	1995	0.3815	-0.23	13.86	-2.21	0.39	4.48
8	1976	0.3491	-0.6	-5.96	8.7	-1.7	3.27
Mass loss years							
8	1988	-0.2796	0.33	4.28	0.56	2.04	2.82
7	2008	-0.3273	0.3	-13.65	-2.57	-2.67	-0.15
6	2011	-0.3726	0.53	10.33	11.19	0.46	4.84
5	2006	-0.3733	0.15	-24.09	-6.09	-2.15	-1.18
4	2003	-0.3976	0.2	-11.11	6.03	-2.19	6.06
3	1990	-0.517	0.66	-12.42	-2.48	-0.11	-7.83
2	1999	-0.554	0.95	8.6	3.51	0.81	-2.77
1	2000	-0.6932	0.88	-9.31	1.96	0.65	-7.5

Supplementary Table 5: Difference of means for key climate and regional circulation indices aggregated by upper and lower quintiles of modeled glacier mass balance for the Southern Alps of New Zealand. Bold values show differences that are more than 95% significant, while italicized values are 90-95% significant. Climate indices are Southern Oscillation Index (SOI), Niño 3.4 index (N3.4), M1 index, MZ3 index, Southwest Pacific Blocking (SWPacB)[15], Southeast Pacific Blocking (SEPacB)[15], Southern Annular Mode (SAM), Pacific South American pattern (PSA), Zonal Wave 3 index (ZW3), Tasman Sea sea surface temperature (Tas SSTa), and selected New Zealand synoptic weather types following Kidson[16] termed T (trough), HE (high to the east of New Zealand) H (high), W (westerly). *Ross Sea sea ice time series from National Snow and Ice Data Center was used for this analysis, under the working assumption that it can be employed as an inverse proxy for Northern Victoria Land sea ice concentration.

	Apr-Jun		July-Sept		Oct-Dec		Jan-Mar		Cool Season		Warm Season		Glacier Year	
	Gain	Loss	Gain	Loss	Gain	Loss	Gain	Loss	Gain	Loss	Gain	Loss	Gain	Loss
mean	-0.58		-0.32	0.15	*-0.57*	*0.58*	-0.53	0.44	-0.46	0.00	*-0.55*	*0.51*	-0.50	0.26
stdev	0.90	1.17	1.30	1.10	*1.20*	*0.94*	1.40	0.98	1.06	1.04	*1.26*	*0.93*	1.12	0.89
**SOI**		p=0.42		p=0.45		*p=0.05*		p=0.13		p=0.40		*p=0.08*		p=0.16
mean	0.40	0.16	0.11	-0.11	0.24	-0.37	*0.42*	*-0.52*	0.26	0.02	0.33	-0.44	0.29	-0.21
stdev	0.63	0.62	0.74	0.97	1.31	1.15	*1.10*	*0.91*	0.63	0.72	1.19	1.02	0.87	0.83
**N3.4**		p=0.46		p=0.62		p=0.34		*p=0.08*		p=0.49		p=0.19		p=0.26
mean	0.79	0.98	1.05	-0.55	1.05	0.04	1.01	0.03	0.92	0.21	1.03	0.04	0.98	0.12
stdev	3.25	1.98	2.44	3.17	3.25	1.73	2.72	2.68	1.85	1.98	2.83	1.66	2.01	1.4
**M1**		p=0.89		p=0.28		p=0.45		p=0.48		p=0.47		p=0.41		p=0.34
mean	0.19	0.31	0.20	-0.06	0.03	-0.23	0.25	-0.31	0.06	0.10	0.13	-0.27	0.16	-0.07
stdev	0.61	0.50	0.45	0.77	0.87	0.38	0.92	0.59	0.24	0.19	0.80	0.38	0.47	0.23
**MZ3**		p=0.68		p=0.42		p=0.45		p=0.17		p=0.72		p=0.22		p=0.23
mean	-0.07	-0.11	-0.14	-0.10	0.05	-0.05	0.04	-0.07	-0.10	-0.11	0.04	-0.06	-0.03	-0.08
stdev	0.30	0.36	0.36	0.39	0.37	0.15	0.31	0.25	0.23	0.31	0.31	0.14	0.23	0.13
**SW PacB**		p=0.81		p=0.83		p=0.49		p=0.45		p=0.94		p=0.42		p=0.60
mean	0.13	0.00	0.15	-0.11	0.19	-0.17	**0.24**	**-0.14**	0.14	-0.05	*0.22*	*-0.16*	0.18	-0.10
stdev	0.59	0.61	0.36	0.54	0.51	0.40	**0.42**	**0.27**	0.39	0.51	*0.44*	*0.28*	0.34	0.35
**SE PacB**		p=0.67		p=0.28		p=0.14		**p=0.05**		p=0.42		*p=0.06*		p=0.13
mean	-0.13	-0.25	*0.15*	*-0.43*	-0.41	-0.16	-0.53	-0.17	0.01	-0.34	-0.47	-0.16	-0.23	-0.25
stdev	0.82	0.92	*0.7*	*0.58*	0.59	0.62	0.49	0.47	0.65	0.70	0.45	0.47	0.35	0.47
**SAM**		p=0.77		*p=0.09*		p=0.42		p=0.16		p=0.31		p=0.20		p=0.92

mean	**-0.30**	**0.40**	**-0.41**	**0.65**	**-0.19**	**0.56**	**-0.39**	**0.61**	**-0.36**	**0.53**	**-0.29**	**0.59**	**-0.32**	**0.56**
stdev	**0.55**	**0.61**	**0.46**	**0.81**	**0.67**	**0.67**	**0.54**	**0.49**	**0.41**	**0.58**	**0.55**	**0.50**	**0.37**	**0.49**
**PSA**		**p=0.03**		**p<0.01**		**p=0.04**		**p<0.01**		**p<0.01**		**p<0.01**		**p<0.01**
mean	-0.35	0.15	**-0.69**	**0.26**	**-0.38**	**0.38**	**-0.43**	**0.29**	**-0.52**	**0.20**	**-0.41**	**0.34**	**-0.46**	**0.27**
stdev	0.81	0.42	**0.73**	**0.36**	**0.58**	**0.54**	**0.17**	**0.63**	**0.75**	**0.28**	**0.34**	**0.47**	**0.40**	**0.33**
**ZW3**		p=0.14		**p<0.01**		**p=0.02**		**p<0.01**		**p=0.02**		**p<0.01**		**p<0.01**
mean	**-0.32**	**0.23**	**-0.34**	**0.28**	**-0.30**	**0.52**	**-0.39**	**0.20**	**-0.33**	**0.26**	**-0.34**	**0.36**	**-0.34**	**0.31**
stdev	**0.32**	**0.32**	**0.22**	**0.21**	**0.20**	**0.60**	**0.24**	**0.47**	**0.24**	**0.25**	**0.15**	**0.33**	**0.15**	**0.24**
**Tas. SSTa**		**p<0.01**		**p<0.01**		**p<0.01**		**p=0.01**		**p<0.01**		**p<0.01**		**p<0.01**
mean	**-289.60**	**127.74**	*-124.25*	*117.47*	*-74.72*	*153.06*	-182.20	96.51	**-206.93**	**122.60**	*-197.02*	*150.08*	**-162.10**	**135.85**
stdev	**441.04**	**196.68**	*332.29*	*179.41*	*181.08*	*251.93*	308.35	384.92	**378.89**	**154.26**	*343.51*	*295.25*	**125.01**	**201.73**
***Sea Ice**		**p=0.03**		*p=0.10*		*p=0.07*		p=0.15		**p=0.04**		*p=0.06*		**p=0.01**
mean	**0.32**	**-0.38**	**0.29**	**-0.33**	-0.25	-0.02	**0.27**	**-0.40**	**0.30**	**-0.35**	0.01	-0.21	**0.16**	**-0.28**
stdev	**0.56**	**0.50**	**0.68**	**0.38**	0.60	0.75	**0.47**	**0.32**	**0.49**	**0.40**	0.41	0.51	**0.33**	**0.33**
**T**		**p=0.02**		**p=0.04**		p=0.51		**p<0.01**		**p=0.01**		p=0.36		**p=0.02**
mean	-0.11	-0.04	**-0.50**	**0.39**	-0.02	0.11	-0.06	-0.02	**-0.30**	**0.18**	-0.04	0.05	-0.17	0.11
stdev	0.50	0.56	**0.35**	**0.64**	0.88	0.69	0.30	0.56	**0.35**	**0.45**	0.41	0.48	0.32	0.34
**HE**		p=0.97		**p<0.01**		p=0.75		p=0.86		**p=0.03**		p=0.69		p=0.11
mean	-0.26	0.19	-0.07	0.15	-0.12	0.00	-0.22	0.02	-0.17	0.17	-0.17	0.01	**-0.17**	**0.09**
stdev	0.46	0.58	0.73	0.58	0.57	0.73	0.75	0.44	0.32	0.46	0.43	0.36	**0.17**	**0.25**
**H**		p=0.11		p=0.52		p=0.43		p=0.45		p=0.11		p=0.38		**p=0.03**
mean	-0.13	0.16	**0.24**	**-0.14**	0.30	-0.22	0.16	-0.21	0.05	0.01	*0.23*	*-0.21*	*0.14*	*-0.10*
stdev	0.49	0.37	**0.17**	**0.38**	0.91	0.33	0.53	0.60	0.31	0.29	*0.56*	*0.25*	*0.30*	*0.13*
**W**		p=0.20		**p=0.02**		p=0.15		p=0.21		p=0.79		*p=0.06*		*p=0.06*

Supplementary Table 6: Difference of means between our glacier mass balance model ('standard run') and balance simulations of reference[17]. Mass balance simulations include those forced by both natural and anthropogenic climate forcings (full forcings; FULL) and natural climate forcings alone (NAT). Multi-model means and standard deviations (SD) are shown. Two-sample t tests (Standard run, FULL; Standard run, NAT) are carried out on consecutive 11-year periods. P values are listed at the middle years (e.g. p values in the 1978 row represent a comparison of means from 1973-1983, and so on). Bold values show differences that are more than 95% significant. At the end of the table, p values for t tests are shown for the positive mass balance (1980-2005). These data are plotted in Figure 9.

**Year**	**Standard run (mm w.e.a-^{1})**	**FULL mean (mm w.e.a-^{1})**	**FULL SD (mm w.e.a-^{1})**	**p values (Standard run, FULL)**	**NAT mean (mm w.e.a-^{1})**	**NAT SD (mm w.e.a-^{1})**	**p values (Standard run, NAT)**
1973	-769.5	-694.7	532.8		-246.7	287.4	
1974	-1638.4	-700.4	411.0		-245.4	447.5	
1975	-2175.1	-500.8	330.2		-375.6	441.7	
1976	104.5	-591.3	373.4		-385.6	474.4	
1977	-1107.1	-402.0	383.6		-377.9	399.2	
1978	-755.5	-273.2	379.4	0.07	-263.2	443.9	**0.01**
1979	-1974.1	-442.8	358.5	0.10	-204.1	318.3	**0.02**
1980	-809.7	-525.7	716.7	0.09	-229.4	355.2	**0.03**
1981	-299.6	-711.7	631.8	0.08	-306.7	376.0	**0.02**
1982	-1606.5	-675.4	302.9	**0.01**	-398.0	461.9	**<0.00**
1983	91.0	-368.2	409.0	**0.01**	-476.9	410.6	**<0.00**
1984	-146.3	-377.1	345.3	0.07	-271.2	340.5	**0.01**
1985	-1298.5	-254.8	340.2	0.08	-248.6	322.5	**0.02**
1986	-1924.0	-237.2	450.6	0.09	-144.1	467.4	**0.02**
1987	-1246.6	-283.7	556.8	0.18	166.3	395.5	**0.05**
1988	-1248.4	-536.3	474.6	0.27	-337.7	460.2	0.08
1989	370.6	-482.9	496.7	0.20	-269.6	402.8	**0.04**
1990	-2725.5	-557.4	491.9	0.49	-265.8	414.0	0.13
1991	-689.8	-599.8	495.5	0.60	-325.7	561.1	0.13
1992	549.6	-705.9	483.5	0.97	-257.4	381.0	0.26
1993	-527.0	-597.8	483.6	0.98	-81.7	558.3	0.27
1994	-370.5	-320.2	436.5	0.89	86.9	520.3	0.21
1995	1391.1	-381.0	445.0	0.38	222.3	733.0	0.07
1996	-930.6	-577.0	517.7	0.61	-48.4	489.4	0.10
1997	-229.5	-747.5	431.0	0.42	-282.8	378.8	0.06
1998	-1521.6	-338.3	277.7	0.32	-141.9	480.4	**0.03**
1999	-1726.6	-557.8	409.0	0.39	-95.6	425.4	**0.05**
2000	-3174.1	-556.3	350.3	0.51	-291.2	435.0	0.08
2001	-811.2	-579.8	498.7	0.21	33.1	577.5	**0.01**

2002	-1793.6	-398.8	508.5	0.27	108.8	573.6	**0.01**
2003	-73.9	-721.0	496.9	0.13	-325.8	748.8	**<0.00**
2004	-198.5	-756.4	300.3	0.17	-368.2	297.3	**<0.00**
2005	17.4	-708.3	350.6	0.25	-318.8	601.4	**0.01**
2006	-504.8	-765.3	388.8	0.29	-306.3	523.1	**<0.00**
2007	-904.3	-1026.2	336.3		116.4	399.2	
2008	-1551.9	-744.6	297.7		-249.1	426.4	
2009	-1345.6	-661.9	347.8		11.3	436.4	
2010	-1031.3	-780.7	321.2		-255.5	559.8	
2011	-2569.7	-727.2	643.1		-3.6	555.7	
				t tests (Standard run, FULL)		t tests (Standard run, NAT)	
positive mass balance period (1980-2005)				p = 0.17		**p= 0.01**	

Supplementary Discussion

On first examination of the reanalysis composite figures for near-surface air pressure (z1000) (Supplementary Figure 4), gain seasons/years on average appear spatially similar to conditions associated with negative Southern Oscillation Index (SOI) values (El Niño), while loss years (particularly in spring and summer) look similar to those associated with positive SOI values (La Niña) (Supplementary Figure 4). Likewise, the depiction for regional sea surface temperatures in the south west Pacific for all gain years correspond to positive Niño3.4 temperatures (El Niño) and vice versa for loss years, particularly for the austral warm half of the year (October-March) and component seasons (October-December, January-March) (Supplementary Figure 4).

However, as shown in Supplementary Figure 5, there is no clear separation in the distribution of SOI and Niño3.4 values between gain and loss years. T-tests of the difference of the mean SOI or Niño3.4 index for growth years versus loss years for each seasonal category shows few significant differences at the 95% confidence level; only the austral spring (October-December) gain and loss quintiles for the SOI are close to being significant (p= 0.051). Scatter plots of the indices compared to mass balance changes show no strong groupings or significant linear relationships to either metric of the El Niño Southern Oscillation (ENSO) (Supplementary Figure 5; Supplementary Table 5). For the only near-significant relationship identified (October-December), SOI mean value versus annual mass balance volume changes) correlations are weak (r=0.28; n=16).

No significant differences between gain and loss years/seasons are indicated by the two-sample t-test observed for M1 and MZ3 Trenberth index values or for either blocking index, and limited significant differences are shown for the Southern Annular Mode (only during winter; Supplementary Table 5). An assessment of Kidson synoptic type frequencies for both gain and loss seasons indicated few significant differences for individual synoptic types. One main exception was observed. The 'T' (‘trough’) synoptic type differences for gain and loss years for April-June (p=0.02), July-September (p=0.04), January-March (p=<0.01), cool season (April-September; p=0.01) and Glacier Year (April-following March; p=0.02) are all significant. On a minor note, the 'HE' type was different only for July-September and cool season (p=0.01 and 0.03) and the 'H' and ‘W’ types were only different for

Glacier Year (p=0.03) and July-September (p=0.02), respectively (see Supplementary Table 5).

Zonal Wave 3 shows significant differences between the gain and loss years for a majority of seasons (except southern autumn). The comparison for Pacific South American pattern values shows significant differences between the gain and loss groups for all seasons. Of all the variables and indices analysed, Tasman Sea surface temperature (average from 25 grid points within the 35-45°S; 155-170°E domain) is the only one that exhibits consistently significant differences (>99% confidence) between the gain and loss years for all seasons (Supplementary Table 5).

Tasman Sea surface temperature versus mass balance change consistently shows significant differences for the gain and loss years for all seasons (Supplementary Figure 6). This indicates that local sea surface temperature anomalies directly offshore of the South Island in the central-southern Tasman Sea may be the primary climatological control on Southern Alps temperature, and therefore, mass balance changes for Southern Alps glaciers. Following on from previous work that has indicated Southern Alps glaciers respond to atmospheric circulation at a regional scale[18-22], we undertook comparisons testing for difference of means (as above) using a wide range of local and hemispheric-scale climate indices to assist interpretation of all reanalysis composite patterns (Supplementary Table 5). Prior research suggested ENSO fundamentally controls Southern Alps glacier activity via atmospheric teleconnections that produce atmospheric circulation anomalies near New Zealand on seasonal time scales[23]. However, the observed spread in the SOI and Niño3.4 values (including clear La Niña archetypal patterns associated with mass balance gain seasons/years, see https://speakerdeck.com/spclimateniwa/composite-plots-for-glacier-mass-balance-gain-and-mass-balance-loss-seasons-and-years) suggests a few 'strong' seasons control the spatial expression of the composite reanalysis patterns when all seasons/years are combined. Because of the finding that extreme years are strongly influencing the mean composite pattern, because of the identified spread that is observed for gain and loss seasons associated with opposite ENSO phases, and because of the wide scatter when our modelled glacier volume changes are regressed against both the SOI and Niño3.4 (Supplementary Figure 5), we suggest ENSO is not the primary control on mass balance variability for the Southern Alps. While ENSO is

likely a contributor (via spring atmospheric teleconnections) during some seasons/years to regional drivers of glacier behaviour (i.e. via a non-linear regional and/or local climate response), this contribution has been, and can be, highly variable.

The Pacific South American pattern shares an atmospheric centre of action with ENSO that is located to the south of the central-equatorial Pacific[24,25]. Our analysis shows that the Pacific South American pattern plays an important role in driving New Zealand glacier mass balance in all seasons (Supplementary Figure 6). The association between the Pacific South American pattern and ENSO variability at inter-annual scales (including persistence of low and high pressures in the Amundsen Sea sector in response to La Niña and El Niño) directly generates anti-phased sea surface temperature and sea ice patterns in the Antarctic Dipole western hemisphere region. However, the absence of a strong ENSO relationship (Supplementary Figure 5) suggests the link between ENSO and New Zealand glaciers, presumed to be via an atmospheric teleconnection, is not instantaneous. This advocates for extratropical drivers as being more important for forcing inter-annual variability of Southern Alps glaciers. The Southern Annular Mode shows few significant influences on glacier gain and loss while Zonal Wave 3 influences all seasons except autumn (Supplementary Figure 6). The testing for difference of means and scales of index value anomalies indicates atmospheric circulation contributions from Zonal Wave 3 are strongest in the austral cool season, particularly for glacier mass gain. The input from Zonal Wave 3 during this time aligns to well-recognised climatological patterns[5,26,27].

The significance of the changes in those atmospheric drivers are reflected via an expression of some specific New Zealand synoptic weather types. The observed 'T' synoptic type frequency difference between gain and loss years during winter, summer, the cool season and for the glacier year are all significant. The occurrence of the “T” type may help to advect cooler waters northward into the Tasman Sea, and promote cooler air temperatures[16,28]. The cooler temperatures favour glacier mass gain by increasing the snow component of total precipitation during spring and reducing melt during summer, increasing the length of the accumulation season. Increased snow during spring also increases the glacier albedo, delaying the melt season onset and dramatically reducing melt season length (Supplementary Figure 7).

The North Victoria Land sea ice anomalies may be associated with the development of high latitude cyclones [29], in a location that would lead to low pressure anomalies to the south of New Zealand. These low pressures are consistent with the “T” synoptic type and guide a high-to-mid latitude atmospheric flow that impacts central Tasman Sea surface temperature patterns. Glacier mass gain years are associated with increased sea ice due south of New Zealand off the coast of Northern Victoria Land in Antarctica, and reduced sea ice extending from the eastern Ross Sea sector to the Amundsen Sea (Fig. 8). The sea ice pattern just described is essentially reversed during mass balance gain years.

Our analysis suggests that multiple regional climate drivers work together to modulate Tasman Sea surface temperature anomalies and thereby Southern Alps glacier responses. In particular, the configuration of the Pacific South American pattern, and Zonal Wave 3, together (Fig. 7, Supplementary Figure 6) illustrate how the 'dice were loaded' through the glacier year to support formation and positioning of certain New Zealand synoptic types that influence Tasman Sea surface temperature.

Supplementary References

1 Chinn, T., Fitzharris, B. B., Willsman, A. & Salinger, M. J. Annual ice volume changes 1976–2008 for the New Zealand Southern Alps. *Global and Planetary Change* **92–93**, 105-118, doi:http://dx.doi.org/10.1016/j.gloplacha.2012.04.002 (2012).

2 WGMS. *Global Glacier Changes: Facts and Figures* (United Nations Environmental Programme and WGMS, 2008).

3 QGIS Development Team, 2009. QGIS Geographic Information System. Open Source Geospatial Foundation Project. http://www.qgis.org/.

4 Kistler, R. *et al.* The NCEP–NCAR 50–Year Reanalysis: Monthly Means CD–ROM and Documentation. *Bulletin of the American Meteorological Society* **82**, 247-267 (2001).

5 Raphael, M. N. A zonal wave 3 index for the Southern Hemisphere. *Geophysical Research Letters* **31**, L23212, doi:10.1029/2004GL020365 (2004).

6 Gong, D. & Wang, S. Definition of Antarctic Oscillation index. *Geophysical Research Letters* **26**, 459-462, doi:10.1029/1999GL900003 (1999).

7 Norton, D. A. A multivariate technique for estimating New Zealand temperature normals. *Weather and Climate* **5**, 64-74 (1985).

8 Knap, W. H. O., J. The surface albedo of the Greenland ice sheet: satellite-derived and in situ measurements in the Sondre Stromfjord area during the 1991 melt season. *Journal of Glaciology* **42**, 364-374 (1996).

9 Anderson, B., Lawson, W. J., Owens, I. F. & Goodsell, B. Past and future mass balance of Ka Roimata o Hine Hukatere (Franz Josef Glacier). *Journal of Glaciology* **52**, 597-607 (2006).

10 Chinn, T. J. *Snow and ice balance measurements from the Tasman Glacier, Waitaki catchment, New Zealand*. (Institute of Geological and Nuclear Sciences Client Report 413399.22., 1994).

11 Kirkbride, M. Relationships between temperature and ablation on the Tasman Glacier, Mount Cook National Park, New Zealand. *New Zealand Journal of Geology and Geophysics* **38**, 17-27 (1995).

12 Purdie, H. *et al.* Interannual variability in net accumulation on Tasman Glacier and its relationship with climate. *Global and Planetary Change* **77**, 142-152, doi:http://dx.doi.org/10.1016/j.gloplacha.2011.04.004 (2011).

13 Chinn, T. *Glacier inventory of New Zealand*. (Institute of Geological and Nuclear Sciences, 1991).

14 Pfeffer, W. T. *et al.* The Randolph Glacier Inventory: a globally complete inventory of glaciers. *Journal of Glaciology* **60**, 537-552, doi:10.3189/2014JoG13J176 (2014).

15 Renwick, J. A. & Revell, M. J. Blocking over the South Pacific and Rossby Wave Propagation. *Monthly Weather Review* **127**, 2233-2247, doi:10.1175/1520-0493(1999)127<2233:BOTSPA>2.0.CO;2 (1999).

16 Kidson, J. W. An analysis of New Zealand synoptic types and their use in defining weather regimes. *International Journal of Climatology* **20**, 299-316 (2000).

17 Marzeion, B., Cogley, J. G., Richter, K. & Parkes, D. Attribution of global glacier mass loss to anthropogenic and natural causes. *Science* **345**, 919-921, doi:10.1126/science.1254702 (2014).
18 Chinn, T. J. Glacier fluctuations in the Southern Alps of New Zealand determined from snowline elevations. *Arctic and Alpine Research* **27**, 187-198 (1995).
19 Fitzharris, B., Chinn, T. & Lamont, G. Glacier balance fluctuations and atmospheric circulation patterns over the Southern Alps, New Zealand. *International Journal of Climatology* **17**, 745-763 (1997).
20 Lamont, G. N., Chinn, T. J. & Fitzharris, B. B. Slopes of glacier ELAs in the Southern Alps of New Zealand in relation to atmospheric circulation patterns. *Global and Planetary Change* **22** (1999).
21 Hooker, B. L. & Fitzharris, B. B. The correlation between climatic parameters and the retreat and advance of Franz Josef Glacier, New Zealand. *Global and Planetary Change* **22**, 39-48 (1999).
22 Chinn, T. J., Winkler, S., Salinger, M. J. & Haakensen, N. Recent glacier advances in Norway and New Zealand: A comparison of their glaciological and meteorological causes. *Geografiska Annaler* **87 A**, 141-157 (2005).
23 Mullan, B. On the linearity and stability of Southern Oscillation-Climate relationships for New Zealand. *International Journal of Climatology* **15**, 1365-1386 (1995).
24 Mo, K. C. Relationships between Low-Frequency Variability in the Southern Hemisphere and Sea Surface Temperature Anomalies. *Journal of Climate* **13**, 3599-3610, doi:10.1175/1520-0442(2000)013<3599:RBLFVI>2.0.CO;2 (2000).
25 Mo, K. C. & Paegle, J. N. The Pacific–South American modes and their downstream effects. *International Journal of Climatology* **21**, 1211-1229, doi:10.1002/joc.685 (2001).
26 van Loon, H. & Jenne, R. L. The zonal harmonic standing waves in the Southern Hemisphere. *Journal of Geophysical Research* **77**, 992–1003 (1972).
27 Fogt, R. L., Jones, J. M. & Renwick, J. Seasonal Zonal Asymmetries in the Southern Annular Mode and Their Impact on Regional Temperature Anomalies. *Journal of Climate* **25**, 6253-6270, doi:10.1175/JCLI-D-11-00474.1 (2012).
28 Renwick, J. A. Kidson’s synoptic weather types and surface climate variability over New Zealand. *Weather and Climate* **31**, 3-23 (2011).
29 Yuan, X., Martinson, D. G. & Liu, W. T. Effect of air-sea-ice interaction on winter 1996 Southern Ocean subpolar storm distribution. *Journal of Geophysical Research: Atmospheres* **104**, 1991-2007, doi:10.1029/98JD02719 (1999).
